# Incidence and Predictors of Severe Adverse Drug Reactions among Patients on Antiretroviral Drugs in Harari Regional State, Eastern Ethiopia

**DOI:** 10.1155/2024/5580728

**Published:** 2024-01-19

**Authors:** Obsa Anbessa, Behailu Hawulte, Tariku Dingeta, Abdi Birhanu

**Affiliations:** ^1^LonAdd Consultancy Plc Seconded by UNICEF at Harari Regional Health Bureau, Harar, Ethiopia; ^2^School of Public Health, College of Health and Medical Science, Haramaya University, Harar, Ethiopia; ^3^School of Medicine, College of Health and Medical Science, Haramaya University, Harar, Ethiopia

## Abstract

**Background:**

The introduction of combination antiretroviral therapy improves the quality and longevity of people living with HIV/AIDS. However, adverse drug reactions associated with antiretroviral therapy compromise the resulting benefits and have been reported differently worldwide, including Ethiopia. Severe adverse drug reactions are one of the major public health concerns for the reason that they can potentially impede the benefit of antiretroviral therapy and put the patient's survival at risk. Despite many successes achieved with the introduction of the combined antiretroviral therapy, the majority of the patients on antiretroviral therapy experience adverse drug reactions associated with the drugs. Consequently, little is known about the problem in the current study area. This is, therefore, to study incidence and predictors of severe adverse drug reactions among patients on antiretroviral drugs in the Harari region, Eastern Ethiopia. The aim of this study was to assess the incidence and predictors of severe adverse drug reactions among patients on antiretroviral therapy from February 25, 2022, to March 25, 2022, in the Harari region, Eastern Ethiopia.

**Methods:**

A hospital-based retrospective cohort study was conducted among 449 randomly selected medical records of people living with HIV on first-line antiretroviral therapy. Collected data were entered into EpiData version 3.1 and exported to STATA version 15 for analysis. Kaplan–Meier survival curve with log-rank test was used to compare survival curves for categorical independent variables. A *p* value ≤0.05 was declared as significant, and an adjusted hazard ratio was used to report the effect size using the multivariate Cox proportional hazard model.

**Result:**

The overall incidence density of the severe adverse reactions was 7.22 per 1000 months (95% CI: 5.5, 9.6). After adjusting for all potential confounders using multivariable Cox proportional hazard ratio, advanced clinical diseases (AHR = 3.44; 95% CI: 1.54, 7.65), HIV/tuberculosis confections (AHR = 2.38; 95% CI: 1.23, 4.62), and being female (AHR = 3.12; 95% CI: 1.57, 6.18) were significantly associated with the experience of severe adverse drug reactions.

**Conclusion:**

In this study, the incidence of severe adverse reactions was consistent with the previous studies, and advanced World Health Organization (WHO) clinical stage, HIV/TB confection, and being female were the independent predictors of the severe adverse drug reactions.

## 1. Introduction

Human immunodeficiency virus (HIV) infected more than 79 million people and killed more than 36 million lives since the start of its epidemics. It remains one of the most serious global health threats of our time [1, 2]. Currently, 37.7 million people are living with HIV (PLWH) globally. In Ethiopia, 620,000 people were living with HIV of which 78% were on ART at the end of 2020 with an estimated prevalence of 0.9% nationally [3].

The introduction of antiretroviral therapy significantly modified the natural history of HIV infection and changed it from an end-of-live event to a manageable chronic disease [4]. However, alongside of all its advantages, challenges related to its associated adverse drug reactions (ADRs) are threatening the progress still, which may range from mild to serious life-threatening ADRs with both short- and long-term effects. Although the most recently approved drugs generally show an improved safety profile than first-generation antiretroviral, drug-related ADRs continue to occur. ADRs are one of the major constraints of the combined antiretroviral therapy (cART) among patients receiving cART, and it is a very common complication of the cART therapy and a major reason for defaulting during the treatment [5].

An adverse drug reaction is a harmful event or unpleasant reaction, attributed to an intervention related to the use of a medicinal product, which predicts hazards from future administration and warrants prevention or specific treatment, or alteration of the dosage regimen, or withdrawal of the product [6]. The ADRs in resource-limited settings may diverge from those developed countries because of different reasons such as the high prevalence of TB, reliance on traditional medicines, and malnutrition and poverty, and also patients come with advanced stages of the disease [7–10]. The adverse events/toxicity associated with cART drugs are more likely to lead to the drug switch among the patients on the treatment, negative impact on the public health system, and poor adherence to the treatment which may result in negative clinical outcomes [11, 12].

Adverse drug reaction surveillance (drug safety surveillance) was established in Ethiopia in 2002 under the Food, Medicine and Health Care Administration and Control Authority currently named as Food and Drug Authority when the country became a member of the WHO program for international drug monitoring. The federal regulatory authority is responsible and empowered with monitoring and ensuring the postmarket drug safety, enabling all issues with ADR. Despite this, the organization collected and documented only a few reports from the health facilities due to different reasons. As the case report analysis conducted showed there is a significant decrease in ADR reporting from 2016 to 2018 which does not show the absence of ADR, majority of the healthcare professionals were not aware of the existence of the spontaneous ADR reporting system which shows a low response and lack of knowledge at ground level [13–17].

To overcome the adverse drug reactions associated with antiretroviral therapy (ART), different intervention was undertaken of which Stavudine (d4T) which was one of the first-line ART drugs was recommended by WHO to phase out from the first-line regimens due to its well-known long-term mitochondrial toxicity [18, 19]. Ethiopia also amended its guidelines to initiate newly diagnosed patients on non-d4T regimens starting from 2012 and other patients gradually as recommended by WHO [20]. Several previous studies on adverse reactions of ART were focused on the incidence and predictors of all forms of adverse reactions, and few assessed the severe forms of the adverse reactions of ART drugs in Ethiopia, particularly in the current study area. Thus, the current study aimed to assess the incidence and its predictors of severe adverse reactions among patients with advanced WHO clinical stage as exposed and those with nonadvanced WHO clinical stage as unexposed at baseline in Harari, Eastern Ethiopia, from January 1, 2018, to December 2020.

## 2. Materials and Methods

### 2.1. Study Design, Setting, and Period

This study was conducted in public hospitals of Harari Regional National State, Eastern Ethiopia. Harari region is located in the Eastern part of Ethiopia at a distance of 526 km away from the capital city Addis Ababa/Finfinnee. The region has a total population of 276,424. The availability of health facility coverage in the region is more than 100%, having 4 hospitals (1 private, 2 public, and 1 governmental hospital), 8 health centers, and 2 not-for-profit nongovernmental organization (NGO) health facilities (HFs) (one fistula center and sexual reproductive health (SRH) center/clinic). Currently, there are a total of 9 ART sites (4 hospitals, 4 urban health centers, and 1NGO clinic) with a total of 4,224 HIV patients on ART in the region. Randomly, selected public health hospitals were the study setup [21]. The data extraction was conducted from February 25 to March 25, 2022.

### 2.2. Study Population and Eligibility Criteria

The study populations were PLWH patients attending ART clinics in Harari region hospitals. The study cohort assigned the study participants into the exposed group (those with advanced WHO clinical stage (III & IV) at baseline) and the unexposed group (those with unadvanced WHO clinical stage (I & II) at baseline). The eligibility criteria were all adult (age 15 and above) HIV patients who were on ART and enrolled from January 2018 to December 31, 2020, at the study setups and on follow-up with at least one follow-up/repeated visit and those with complete information about severe ADRs.

### 2.3. Sampling Technique, Procedure, and Sample Simple Calculation

The sample size of the study was determined for survival data, Cox proportional regression model by using Epi Info 7.0 by taking the percentage of exposed group and unexposed group who experienced severe adverse drug reactions 19% and 9%, respectively, from previous study conducted in Debre Markos referral hospital [22], with the consideration of the following assumption margin of error 5% and 95% confidence interval, power 80%, and by adding 10% of the loss to follow-up which gives 150 of exposed group and 299 of unexposed group and total sample size of 449 with a ratio of exposed to unexposed group 1 : 2. The study settings were selected randomly, and lists of all eligible participants were obtained from ART clinics in the selected facilities and were used as the sampling frame. The patient's ART unique identification number (ID) was used to select patients' medical record using computer-generated simple random sampling techniques.

### 2.4. Study Variables and Measurement

Severe adverse drug reactions were the outcome variable. The severe adverse drug reactions were defined as documented on the ART chart/follow-up chart, history sheet, or progress note by the ART nurse or physician. Independent/exposure variables were subgrouped under three thematic areas: sociodemographic characteristics are the pretreatment demographic characteristics such as age, sex, place of residence, occupational status, educational status, marital status, and religion and enrollment month/year. Behavioral characteristics are disclosure status. Clinical and immunological characteristics are body mass index (BMI), CD4 count, WHO clinical stage, the experience of tuberculosis (TB) infection, experience of other opportunistic infections (OIs), experience of anti-TB prophylaxis, experience of regimen change, functional status, experience of taking other medication/nutritional supplements, baseline regimen, cotrimoxazole therapy (CPT), and baseline nutritional status. The study participants were categorized as exposed and unexposed groups based on the baseline WHO clinical stage of HIV/AIDS. Patients with WHO clinical stages III and IV and I and II were classified as exposed and unexposed groups, respectively.

### 2.5. Data Collection and Quality Control

A structured data extraction form was developed from ART follow-up charts and relevant literature and used [22–25]. Three clinical nurses were deployed for data collection. One BSC nurse who received a training on ADR in ART was assigned to supervise and coordinate the data collection processes. A two-day training on the objective of the study, data collection tool, and extraction technique was given by the authors. The supervisor followed the day-to-day data collection activities to make sure that the collected data are complete, consistent, and accurate.

### 2.6. Data Processing and Statistical Analysis

Data were entered using EpiData version 3.1 and exported to STATA for cleaning and analysis. All randomly selected study participants were followed for a maximum of 36 months (from January 2018 to December 31, 2020), and a total of 449 study participants were included in the analysis. Patients who lost to follow-up and transferred out after at least repeated follow-up and died before experiencing the events were considered as censored during analysis. All patients were followed until the occurrence of an event of interest (severe adverse drug reaction) or censored. Descriptive statistics such as frequency, percentage, mean, and standard deviation (SD) were calculated. Kaplan–Meier (KM) survival curve with log-rank test was used to compare survival curves for categorical independent variables. The median and mean survival time were estimated using the KM method. To predict the association between independent and outcome/dependent variables, the Cox proportional hazard regression model was used. 95% CI of the hazard ratio was calculated, and the variable with *p* value ≤ 0.05 in the Cox proportional hazards model was considered and declared as significant. For the evaluation of the proportional hazard assumption, log-log survival curves and Schoenfeld's goodness-of-test approach were used. A life table was used to calculate the cumulative survival probability of the patients. Adjusted hazard ratio was used to report the effect size. The Schoenfeld residuals and global test were used to check the goodness-of-model fitness.

### 2.7. Ethical Statement

Ethical clearance was obtained from the Institutional Health Research Ethics Review Committee (IHRERC) with ref. no. IHRERC/026/2022 of the College of Health and Medical Science, Haramaya University Health and Medical Science College. An ethics approval letter from IHRERC was delivered to study facilities in the Hiwot Fana Comprehensive Specialized University Hospital and Jugol Hospital. Final approval for data extraction was obtained from study facilities. Informed, voluntary, written, and signed consents were obtained from each head of the respective health facility before data collection was started. As the study involves retrospective data, there was no direct harm to the patients. To ensure the confidentiality of the patients' records, data extracted from the patient follow-up charts were deidentified and stored in a secured computer which was only accessible to authors and supervisors.

## 3. Results

### 3.1. Sociodemographic Characteristics

A total of 735 HIV-positive patients were enrolled in the ART program in two facilities (Hiwot Fana 573 and Jugol 162) from January 2018 to December 2020. In this study, a total of 449 (150 exposed and 299 unexposed) participants' data were retrieved and included in the analysis of which 55.5% of the participants were females. Nearly half of the patients' ages ranged from 35 to 44 years at the time of ART initiation with a mean (+standard deviation) age of 38 (+8.44) years. Nearly one-third of the participants were self-employed, and 58.8 percent of the clients were married. More than one-third of 67.04% of the study participants came out of the catchment. Concerning the educational level and religion of the patients, 168 (37.42%) and 261 (58.13%) patients completed primary education and are Orthodox Christian followers, respectively (Table 1).

### 3.2. Behavioral Characteristics

Of a total of 449 (150 exposed and 299 unexposed), 67.26% disclosed their sero-status to family members or sexual partners and 30.8% were not disclosed yet.

### 3.3. Clinical Characteristics

From a total of 449 (150 exposed and 299) HIV-positive patients on ART follow-up, 80.62% had a CD4 count of more than or equal to two hundred cells/mm^3^ and more than one-third of the patients had normal BMI during ART initiation. Most of the HIV-positive patients at ART initiation time were WHO clinical stage I (39.2%), around 15% of the patients experienced HIV/TB infection at baseline, one-third of the patients were on 1e (TDF-tenofovir + 3TC-lamivudine + EFV-efavirenz) regiment at baseline, and more than half of the patients experienced regimen change. More than three-fifth of 61% of the participants were on working functional status during the ART initiation, and 80% of the patients had good adherence to ART. More than one-third of the patient's nutritional status was normal at the time of ART initiation (Table 2).

### 3.4. Survival Probability

Due to the smaller events that occurred in the cohort, the median survival time was not computable. About 40 (81.63%) severe adverse drug reactions occurred in the first year of ART enrollment, of which 29 (59.1%) occurred in the first six months of ART enrollment. The cumulative survival probability of developing severe ADRs of the HIV-positive patients on ART at six, twelve, twenty-four, and thirty-six months was 0.82, 0.57, 0.23, and 0.036, respectively (Table 3).

**Figure 1:**
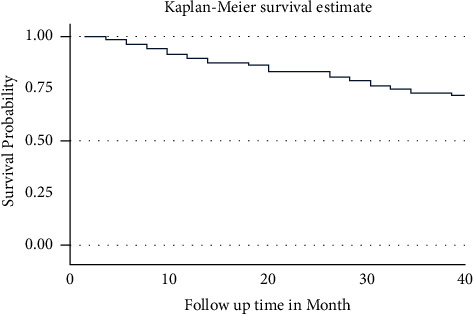
Overall Kaplan–Meier survival estimate curve among PLWHA on ART in Harari, Eastern Ethiopia.

The overall survival probability of the patients through the treatment time decreased as the time increased (Figure 1).

**Figure 2:**
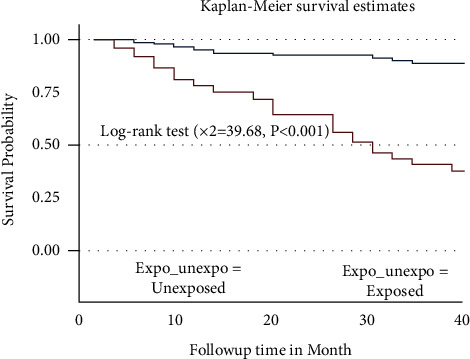
Kaplan–Meier survival estimate curve for the time to develop severe ADRs among PLWHA on ART by their exposure status in Harari, Eastern Ethiopia.

The survival probability among the unexposed group is high compared to the exposed group throughout, and the log-rank test indicates a statistically significant survival probability between the two groups (Figure 2).

**Figure 3:**
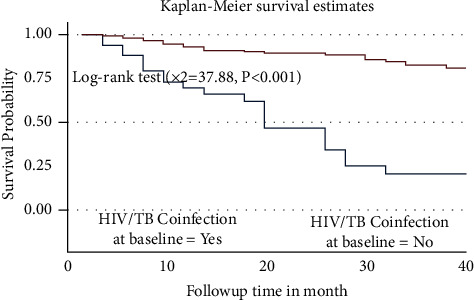
Kaplan–Meier survival estimate curve for the time to develop severe ADRs among PLWHA on ART by HIV/TB coinfection in Harari, Eastern Ethiopia.

The survival probability of PLWH who did not experience TB/HIV confection was high when compared to those who experienced it, and the log-rank test indicates a statistically significant difference in survival between the two groups (Figure 3).

**Figure 4:**
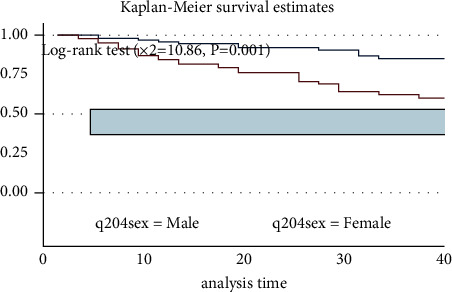
Kaplan–Meir survival estimate curve for the time to develop severe ADRs among PLWHA on ART by gender in Harari, Eastern Ethiopia.

The survival probability of males was high when compared to females, and the log-rank test indicates a statistically significant difference in survival between the two groups of PLWH (Figure 4).

### 3.5. Incidence of Severe ADRs

A total of 449 (150 exposed and 299) retrieved data were followed for the last three years; of these, a total of 49 (10.91%) of them developed severe ADRs in 6782 person months of observations. Incidence density among exposed was 1.8 per 100 person month (95% CI: 0.013, 0.025) and 0.27 (95% CI: 0.16, 0.47) per 100 person month among unexposed. The incidence among females was 1.1 (95% CI: 0.78, 1.5) per 100 person month and 0.34 (95% CI: 0.18, 0.61) per 100 person month. The overall incidence density was 7.22 (95% CI: 5.5–9.6) per 1000 person-month observations. The most commonly reported complaints were hematological problems, gastrointestinal problems, dermatological problems, neurological problems, and others (kidney and liver toxicity) which accounts for 26.6%, 24.5%, 22.4%, 22.4%, and 4.1%, respectively.

### 3.6. Predictors of the Severe ADRs

After proportional hazard assumptions were checked for each covariate, all covariates with a *p* value of ≤0.25 in bivariate analysis (health facility, sex, baseline BMI, WHO clinical stage, HIV/TB confection, experiencing other OIs than TB, and taking other medication at baseline) were considered as a candidate for multivariate Cox proportional hazard regression analysis. In the final model, three variables, gender, WHO clinical stage, and HIV/TB coinfection, were significantly associated with severe ADRs after model fitness was checked with Schoenfeld residual test. Female HIV-positive patients on ART follow-up were about three times (AHR = 3.14 95% CI: 1.59, 6.22) more likely to experience severe ADRs compared to male patients. The risk of experiencing severe ADRs among patients presented with advanced WHO clinical stage (exposed) at baseline was about three times (AHR = 2.96; 95% CI: 1.33, 6.58) as compared to unexposed patients on ART. Patients with TB confections at baseline were double (AHR = 2.11; 95% CI: 1.09, 4.06) times more likely to experience severe ADRs as compared to a patient with no TB infection at baseline (Table 4).

## 4. Discussion

A total of 449 patients with HIV/AIDS who started ART between January 2018 and December 2020 were followed up and included in the analysis, of which 49 patients experienced severe ADRs.

In this study, we found that 10.91% of patients enrolled in the study experienced severe ADRs over a period of 6,782 person-month observations. Being female, advanced clinical diseases and TB/HIV confection were significantly associated with the time to develop severe adverse drug reactions among PLHIV on ART.

The incidence of severe adverse drug reaction was found to be 0.722 (0.55–0.96) per 100 person month of follow-up which is consistent with randomized controlled trial study conducted in Haiti, 11% [26], observational study in Kenya, 13% [27], observational study in Black Lion Hospital, Addis Ababa, 7.7% [28], Arbaminch, Southern Ethiopia, 0.53 per 100 person month [29], and seven teaching hospital in Ethiopia, 9.5% [25].

On the other hand, the findings of this study were lower than the study conducted in India, 23.1% [30], Nigeria, 16.8% [31], Northern and Southern Ethiopia, 3.2/100 person month [32], and 22.9% [33], respectively. This variability may be due to differences in the follow-up period, which may affect the occurrence of adverse events in HIV patients on ART. In our case, the follow-up period was three years, while the follow-up period was six months in a study conducted in India [30] and 12 months in a prospective study conducted in Ethiopia [32] as ART-associated adverse drug reactions during the first six months of treatment are high/common [25, 34–37]. In addition, data collection methods or study design also may influence the incidence of the ADR. Furthermore, the difference in ADR definition among those studies is a possible reason. For instance, studies in India and in Southern Ethiopia defined ADRs as the occurrence of undesirable effects registered on patient follow-up charts such as nausea, diarrhea, anemia, abdominal pain, skin rash, and anemia, but in our case, severe ADRs were defined as serious/life-threatening reactions which consist of grade 3 and 4 of WHO ADR classification [18]. Another possible reason is that the sample size of the present study is relatively larger than that of the study conducted in India, Northern Ethiopia, and Southern Ethiopia.

In this study, the incidence of severe ADRs is relatively higher than in the two studies conducted in the Northern parts of Ethiopia Tigray (3.6 per 100 person year) [24] and Amhara region (3 per 100 person-years) [22]. This difference may relate to the difference in follow-up time in which the follow-up time in our case is lower than that of the two studies. As the follow-up time increases, the denominator increases which implies a decrease in incidence and the ADRs are likely to occur in the early treatment life [34, 35].

In the current study, the exposed group, those who presented with the advanced clinical disease at ART initiation time (AHR = 3.29; 95% CI: 1.47, 7.39), is three times more likely to experience severe ADRs compared to those unexposed. This finding is supported by an observational study conducted in Nigeria [11], Debre Markos, Northern Ethiopia, Arbaminch, Southern Ethiopia, Kaffa Zone, and Jimma, South West Ethiopia [22, 23, 29, 36]. Patients with advanced disease are more likely to have various comorbidities that can lead to multiple medications being taken at the same time. Patients on multiple medications or polypharmacy are more likely to experience serious side effects due to overlapping toxicities. Patients with advanced disease may also have altered pharmacodynamics and pharmacokinetic mechanisms due to drug reactions that predispose patients to adverse events [37–40]. To address new HIV infections and early ART initiation, Ethiopia initiated case-based surveillance integrated with the testing of index cases and added to notifiable diseases. However, the available information has not yet been well analyzed, reported or presented to the scientific community. This surveillance system uses the hybrid model consisting of paper-based and electronic reporting in which there was nonalignment between them. Behavioral and technical factors such as poor documentation practice, skill gap, and lack of experience which result in poor data quality and poor information utilization for decision-making were the other challenges for the surveillance system in Ethiopia. Moreover, the challenges to the surveillance of low- and middle-income countries including Ethiopia are limited resources, both financial and capacitated human resources [41–45].

Patients experiencing HIV/TB confection at ART initiation were about two times more likely to experience severe ADRs (AHR = 2.35, 95%: 1.22, 4.54) when compared to those who did not experience them. This finding is similar to study findings in seven teaching hospitals in Ethiopia and southern Ethiopia [25, 33]. This could be explained by the overlapping toxicities of anti-TB and ART drugs, which have been well documented, and even some literature delay the initiation of ART during the intensive phase of the TB treatment course. There is also the documented drug-to-drug reaction of the anti-TB and ART drugs [39, 46–49].

In this study, female patients were three times (AHR = 3.06; 95% CI: 1.54, 6.06) at higher risk of experiencing severe ADRs at any time compared to males. This finding is consistent with the study conducted in India [50], Nigeria [31], Ghana [51], Arbamanchi, and southern Ethiopia [29]. Possible explanations may include physiological differences in women having a lower lean body mass than men, reduced hepatic clearance in women, and differences in cytochrome P450 (CYP) enzyme activity and metabolism at different rates compared to men. Another possible explanation is a higher concentration of the drug in the blood and a longer elimination time in women than in men. In addition, women may use birth control and hormone supplements during their reproductive years, which increases the number of medications that can increase the likelihood of ADRs [37, 39, 52, 53].

Being a retrospective study has its limitations since it is based on secondary data. The event documentation may be different depending on the service provider's documentation practice and data utilization culture which leads to underestimation of the event of interest. The other limitation of this study was that the side effects of the ART were not included and reported.

## 5. Conclusion and Recommendation

In this study, the incidence of severe ADRs was relatively consistent with other studies conducted in different parts of the developing and developed countries with a considerable burden. Patients with advanced WHO clinical disease, HIV/TB coinfection, and female patients on the ART initiation should get due attention and close follow-up to minimize the preventable severe ADRs. Routine screening of patients for signs and symptoms of severe ADR is very crucial to prevent timely response and management of it. Additionally, we recommend a prospective study to measure variables more precisely that cannot be addressed through a retrospective study.

## Figures and Tables

**Table 1:** Sociodemographic characteristics of the patients on ART follow-up in Harari, Eastern Ethiopia, and February 25 to March 25, 2022.

Characteristic (*n* = 449)		Exposed (*n* = 150)	Unexposed (*n* = 299)
Hospitals	Hiwotfana Comprehensive Specialized University Hospital (HCSUH)	92 (61.3%)	232 (77.6%)
	Jugol Hospital	58 (39.7%)	67 (22.4%)

Gender	Male	59 (39.3%)	141 (47.2%)
	Female	91 (60.7%)	158 (52.8%)

Age in years	15–24	8 (5.3%)	10 (3.3%)
	25–34	40 (27%)	98 (32.8)
	35–44	68 (46%)	135 (45.2%)
	≥45	34 (21.7%)	56 (18.7%)

Marital status	Single	16 (10.7%)	24 (8%)
	Married	75 (50%)	189 (63.2%)
	Separated/divorced	39 (26%)	64 (21.4%)
	Widowed/widower	20 (13.3%)	22 (7.4%)

Occupation	Employed	33 (22%)	46 (15.4%)
	Daily laborer	20 (13.3%)	39 (13%)
	Driver	11 (7.3%)	31 (10.4%)
	Private	44 (29.3%)	96 (32.1%)
	No job	42 (28%)	87 (29.1%)

Education	No formal education	34 (23%)	85 (28.4%)
	Primary (1–8)	57 (38%)	111 (37.1%)
	Secondary (9–12)	37 (25%)	61 (20.4%)
	College and above	22 (14%)	42 (14.1%)

Residence	Within catchment	49 (33%)	99 (33.1%)
	Out of catchment	101 (67%)	200 (66.9%)

Religion	Orthodox	89 (59.3%)	172 (57.5%)
	Muslim	53 (35.3%)	109 (36.5%)
	Protestant	8 (5.4%)	18 (6%)

**Table 2:** Baseline characteristics and follow-up measurements of patients on ART in Harari, Eastern Ethiopia, February 25 to March 25, 2022.

Characteristics (*n* = 449)		Exposed (*n* = 150)	Unexposed (*n* = 299)
CD4 count (cells/mm^3^)^*∗*^	<200	63 (42%)	24 (8%)
	≥200	87 (58%)	275 (92%)

BMI	Underweight	62 (41.3%)	59 ((19.7%)
	Normal	84 (56%)	226 (75.6%)
	Overweight and obese	4 (2.7%)	14 (4.7%)

Nutritional status	Undernutrition	62 (41.3%)	58 (19.4%)
	Normal	84 (56%)	227 (75.9%)
	Overnutrition	4 (2.6%)	14 (4.6%)

WHO clinical stage	Stage I		176 (58.8%)
	Stage II		123 (41.2%)
	Stage III	128 (85.3%)	
	Stage IV	22 (14.7%)	

Experience of TB infection	Yes	56 (37.3%)	12 (4%)
	No	94 (62.7%)	287 (96%)

Experience of regimen change	Yes	73 (48.7%)	176 (58.8%)
	No	77 (51.3%)	123 (41.2%)

Experience of other OIs other than TB	Yes	92 (61.3%)	84 (28.1%)
	No	58 (38.7%)	215 (71.9%)

Functional status during ART initiation	Working	15 (10%)	257 (86%)
	Ambulatory	106 (70.6%)	42 (14%)
	Bedridden	29 (19.4%)	0

Baseline regimen	1c/1e	109 (72.6%)	215 (71.9%)
	1J	33 (22%)	72 (24%)
	Others^¶^	8 (5.4%)	12 (4.1%)

Experience of taking other medication^¥^	Yes	150 (100%)	217 (72.6%)
	No	0	82 (21.4%)

Ever took CPT	Yes	82 (54.6%)	148 (49.5%)
	No	68 (46.4%)	151 (50.1%)

Ever took anti-TB prophylaxis	Yes	49 (32.6%)	158 (52.8%)
	No	101 (73.4%)	141 (47.2%)

Adherence status	Good	111 (76.6%)	246 (82.6%)
	Fair	26 (18%)	44 (15.1%)
	Poor	7 (5.4%)	7 (2.3%)

^*∗*^CD4 classification is based on the previous studies, 1c = AZT (zidovudine)+3TC + NVP (nevirapine), 1e = TDF + 3TC + EFV, 1J = TDF + 3TC + DTG (dolutegravir), ^¶^other regimens include 1d = AZT + 3TC + EFV, 1g = ABC (abacavir) + 3TC + EFV, 1f = TDF + 3TC + NVP, and 1h = ABC + 3TC + NVP, and ^¥^other medications include medications given for the PLWH other than anti-TB and CPT, therapeutic food, and any analgesics (antipain).

**Table 3:** Life table for severe adverse drug reactions among HIV-positive patients on ART in Harari, Eastern Ethiopia, February 25 to March 25, 2022.

Interval^*∗*^		Beg	Total deaths	Lost	Survival	Error	95% conf. int.	
1	6	449	81	0	0.8196	0.0181	0.7808	0.8522
7	12	342	85	0	0.5724	0.0233	0.5252	0.6166
13	18	228	66	0	0.3608	0.0227	0.3165	0.4052
19	24	146	44	0	0.2272	0.0198	0.1896	0.2669
25	30	86	43	0	0.0958	0.0139	0.0708	0.1252
31	36	41	25	0	0.0356	0.0087	0.0212	0.0558

^*∗*^Months.

**Table 4:** Multivariate Cox regression analysis of the associations of the predictor variables and time to develop ADRs, February 25 to March 25, 2022.

Variables		ADRs		CHR (95% CI)	AHR (95%)
		Event (*n* = 49)	Censored (*n* = 400)		
Health facility	HFSUH	32 (9.88%)	292 (90.12)	1	1
	Jugola	17 (13.6%)	108 (86.4)	1.69 (0.93, 3.06)	1.24 (0.68, 2.29)

Sex	Male	38 (15.26%)	211 (84.74%)	1	1
	Female	11 (5.5%)	189 (94.5%)	3.16 (1.61–6.18)	3.06 (1.54–6.06)^*∗*^

Place of residence	Within the catchment	21 (14.19%)	127 (85.81%)	1.44 (0.82–2.54)	1.24 (0.74–2.46)
	Out of the catchment	28 (9.3%)	273 (90.7%)	1	1

BMI	Underweight	31 (25.62%)	90 (74.38%)	5.39 (3.01–9.67)	0.91 (0.02–52.13)
	Normal/overweight	18 (5.48%)	310 (94.52%)	1	1

WHO clinical stage	Advanced (III and IV)	36 (24%)	114 (76%)	6.2 (3.28–11.70)	3.29 (1.46–7.39)^*∗*^
	Unadvanced (I and II)	13 (4.35)	286 (95.65%)	1	1

TB infection	Yes	22 (33.33%)	46 (67.67%)	5.12 (2.96–9.15)	2.35 (1.22–4.54)^*∗*^
	No	27 (7.08%)	354 (92.92%)	1	1

Experience of OIs	Yes	28 (15.82%)	149 (84.18%)	2.25 (1.27–3.98)	1.08 (0.56–2.09)
	No	21 (7.72%)	251 (92.28%)	1	1

Experience of other medication	Yes	46 (12.53%)	321 (87.47%)	3.91 (1.21–12.61)	1.56 (0.41–5.91)
	No	3 (3.66%)	79 (96.34%)	1	1

^*∗*^ *p* value less than 0.05 indicates statistical significance in multivariable Cox proportional hazard, and 1 indicates a reference value.

## Data Availability

All data that support the findings of this study are accessible from the corresponding author upon demand.

## Abbreviations

AIDS: Acquired immunodeficiency syndrome
ART: Antiretroviral therapy
AHR: Adjusted hazard ratio
BMI: Body mass index
HIV: Human immunodeficiency virus
OIs: Opportunistic infections
PLWH: People living with HIV
TB: Tuberculosis
WHO: World Health Organization.
